# Novel strains of a pandemic plant virus, tomato spotted wilt orthotospovirus, increase vector fitness and modulate virus transmission in a resistant host

**DOI:** 10.3389/fmicb.2023.1257724

**Published:** 2023-09-29

**Authors:** Senthilraja Chinnaiah, Saurabh Gautam, Benjamin Herron, Fekede Workneh, Charles M. Rush, Kiran R. Gadhave

**Affiliations:** ^1^Texas A&M AgriLife Research, Amarillo, TX, United States; ^2^Department of Entomology, Texas A&M University, College Station, TX, United States; ^3^Department of Plant Pathology, Texas A&M University, College Station, TX, United States

**Keywords:** resistance-breaking strains, western flower thrips, *Orthotospovirus tomatomaculae*, insect vector biology, vector-virus interactions

## Abstract

Tomato spotted wilt orthotospovirus (TSWV) is one of the most successful pandemic agricultural pathogens transmitted by several species of thrips in a persistent propagative manner. Current management strategies for TSWV heavily rely on growing single-gene resistant cultivars of tomato (“*Sw-5b*” gene) and pepper (“*Tsw*” gene) deployed worldwide. However, the emergence of resistance-breaking strains (RB) in recent years has compounded the threat of TSWV to agricultural production worldwide. Despite this, an extensive study on the thrips transmission biology of RB strains is currently lacking. It is also unclear whether mutualistic TSWV-thrips interactions vary across different novel strains with disparate geographical origins. To address both critical questions, we studied whether and how four novel RB strains of TSWV (two sympatric and two allopatric), along with a non-RB strain, impact western flower thrips (WFT) fitness and whether this leads to differences in TSWV incidence, symptom severity (virulence), and virus accumulation in two differentially resistant tomato cultivars. Our findings show that all RB strains increased WFT fitness by prolonging the adult period and increasing fecundity compared to non-RB and non-viruliferous controls, regardless of the geographical origin of strains or the TSWV titers in individual thrips, which were substantially low in allopatric strains. TSWV accumulation in thrips varied at different developmental stages and was unrelated to the infected tissues from which thrips acquired the virus. However, it was significantly positively correlated to that in WFT-inoculated susceptible plants, but not the resistant ones. The TSW incidences were high in tomato plants infected with all RB strains, ranging from 80% to 90% and 100% in resistant and susceptible plants, respectively. However, TSW incidence in the non-RB-infected susceptible tomato plants was 80%. Our findings provide new insights into how novel strains of TSWV, by selectively offering substantial fitness benefits to vectors, modulate transmission and gain a potential epidemiological advantage over non-RB strains. This study presents the first direct evidence of how vector-imposed selection pressure, besides the one imposed by resistant cultivars, may contribute to the worldwide emergence of RB strains.

## 1. Introduction

Tomato spotted wilt orthotospovirus (*Orthotospovirus tomatomaculae*, referred to hereafter as TSWV) is ranked second among the most devastating plant pathogenic viruses across the world (Scholthof et al., 2011). TSWV has a wide host range (over 1,000 plant species from >90 plant families), and it is transmitted by several species of thrips (Thripidae; Thysanoptera) in a persistent propagative manner (Whitfield et al., 2005; Pappu et al., 2009). Thrips cause both direct (via feeding) and indirect (via transmission of TSWV) damage to an array of specialty and staple food crops worldwide. Their cosmopolitan distribution shared a wide host range, and rapid reproductive potential have enabled TSWV to become a pandemic agricultural pathogen over the past century. TSWV has 80–110 nm spherical virions containing tripartite negative/ambisense RNA segments: large (L), medium (M), and small (S), with sizes of 8.9, 4.8, and 2.9 kb, respectively (Adkins et al., 1995).

Besides the management of its vector, the use of resistant cultivars is one of the most effective TSWV management strategies. In the past few decades, two dominant single genes, namely, *Sw-5b* and *Tsw*, offering broad-spectrum resistance against TSWV, were identified from wild relatives and deployed into commercial tomato and pepper cultivars, respectively (Boiteux and De Avila, 1994; Boiteux, 1995; Dianese et al., 2011; De Oliveira et al., 2018). The single-gene resistance is mediated through the interaction of nucleotide-binding leucine-rich repeats (NLR) of the *Sw-5b* gene of tomato with TSWV avirulent (Avr) factor, a non-structural movement protein (Nsm), and that of the *Tsw* gene of pepper with TSWV Avr, a non-structural silencing suppressor (Nss) (Margaria et al., 2004; Peiró et al., 2014). However, tremendous selection pressure on TSWV imposed by widely deployed single-gene resistant cultivars of both tomato and pepper has led to the emergence of novel resistance-breaking (RB) strains of TSWV worldwide. To date, these strains have been reported in several countries, including Italy, Australia, Spain, Hungary, South Africa, Turkey, South Korea, Brazil, and the United States (Aramburu and Marti, 2003; Margaria et al., 2004, 2007; Ciuffo et al., 2005; Sharman and Persley, 2006; Zaccardelli et al., 2008; Fidan and Sari, 2019; Yoon et al., 2021; Almási et al., 2023). In the United States, RB strains have been reported in tomatoes from California and North Carolina (Batuman et al., 2017; Lahre et al., 2023) and in peppers from California (Macedo et al., 2019). Most recently, our lab reported novel strains in tomato and pepper for the first time in Texas (Chinnaiah et al., 2023; Gautam et al., 2023). Despite several reports across the world, to date, no study has investigated the thrips transmission biology of RB strains.

Of all thrips species, western flower thrips (WFT), *Frankliniella occidentalis* (Thripidae: Thysanoptera) is reported to be the most prevalent and efficient vector of TSWV (Wan et al., 2020). WFT typically acquires TSWV from infected plant tissues in the early larval stages (Chatzivassiliou et al., 1999). Once acquired, the virus replicates in the thrips midgut lumen and salivary gland, often resulting in the persistent and highly efficient transmission of TSWV throughout the thrips lifespan. Several studies have explored interactions between thrips and TSWV, in that most showed that the TSWV infection offers fitness benefits to thrips by triggering behavioral response and increasing survival, longevity, and fecundity (Belliure et al., 2004; Maris et al., 2004; Shrestha et al., 2012; Shalileh et al., 2016; Nachappa et al., 2020; Wan et al., 2020), while few have reported reduced thrips fitness (De Angelis et al., 1993; Stumpf and Kennedy, 2005; Ogada et al., 2013), affecting TSWV transmission and spread favorably or otherwise. However, almost all previous studies were based on single, wild-type, or sympatric (originating within the same locality) strains of TSWV, in that none rigorously evaluated whether and how virus accumulation at various stages of thrips development affects overall thrips fitness and transmission. A comprehensive study on the thrips transmission of novel sympatric and allopatric (originating from different geographical locations) RB strains is currently lacking.

Since thrips vectoring is indispensable to the evolutionary success of TSWV as a pandemic pathogen of agricultural crops, understanding their interactions in light of resistance-breaking strains originating from different locations is critical to devising robust TSWV management strategies. This study examined whether and how four novel RB strains of TSWV (two sympatric and two allopatric) along with non-RB control, especially their accumulation at various thrips developmental stages, affect thrips fitness (developmental time and fecundity). Furthermore, we examined whether WFT transmits these strains differently and whether this leads to differences in TSWV incidence, symptom severity (virulence), and virus accumulation in two differentially resistant (one resistant and one susceptible) tomato cultivars.

## 2. Materials and methods

### 2.1. TSWV strains

A total of five different TSWV strains were used in this study. Of these strains, four were resistance-breaking (RB), namely, “Tom-BL1,” “Tom-BL2,” “Tom-CA,” and “Tom-MX,” whereas the fifth was a non-resistance-breaking wild-type strain of TSWV, named “non-RB”. Tom-BL1 and Tom-BL2 were sympatric strains recovered from TSW-symptomatic *Sw-5b*-resistant tomato plants grown in a field trial at Bushland, TX (Chinnaiah et al., 2023). On the contrary, Tom-CA and Tom-MX were allopatric strains recovered from TSW-infected tomato fruits showing characteristic chlorotic ringspot symptoms collected from two different supermarkets in Amarillo, TX. The non-RB strain used in this study was recovered from a susceptible pepper cultivar in College Station, TX, as we were unable to obtain a non-RB strain originating from tomatoes despite our extensive efforts over the past 2 years, which is possibly due to the exclusive or near-exclusive adoption of commercial *Sw-5b*-resistant tomato cultivars, as all TSWV isolates collected by our lab turned out to be resistance-breaking. The resistance-breaking status of all four strains and the lack thereof in a non-RB strain were confirmed through mechanical and thrips inoculation of 3-week-old plants from five resistant cultivars and one susceptible one (control) using TSWV-infected symptomatic tissues from RB/non-RB strains. A detailed description of mechanical inoculation, along with the confirmation of TSWV infection status and *Sw-5b*-resistant status of inoculated plants, is provided in the two first reports of TSWV RB strains from our lab (Chinnaiah et al., 2023; Gautam et al., 2023). Plants inoculated with different strains were maintained in separate insect-proof cages to avoid accidental cross-contamination in the greenhouse at 25°C with a 12-h photoperiod. Young leaves showing characteristic TSW symptoms (systemic infection) across all five different strains were subjected to RNA extraction followed by reverse transcriptase-quantitative PCR (RT-qPCR) to confirm the infection status and quantify the virus titer.

### 2.2. Thrips colony

A virus-free colony of WFT was obtained from Diane Ullman at the University of California, Davis. Thrips at various stages of development were reared on surface-sterilized green bean (*Phaseolus vulgaris*) pods (a brief exposure to 10% sodium hypochlorite followed by repetitive washing with water), which also served as an oviposition site, in a semi-transparent plastic container [14 (*h*) × 18.5 (*d*) cm] with a lid fixed with insect-proof mesh in the center [4 cm (*d)*]. The containers were placed in a laboratory at 25°C and a 16-h photoperiod. Bean pods were changed once every 2 days to provide fresh food. Thrips were reared continuously to obtain numerous first-instar larvae used in the experiment.

### 2.3. Thrips fitness and TSWV accumulation

**Figure 1:**
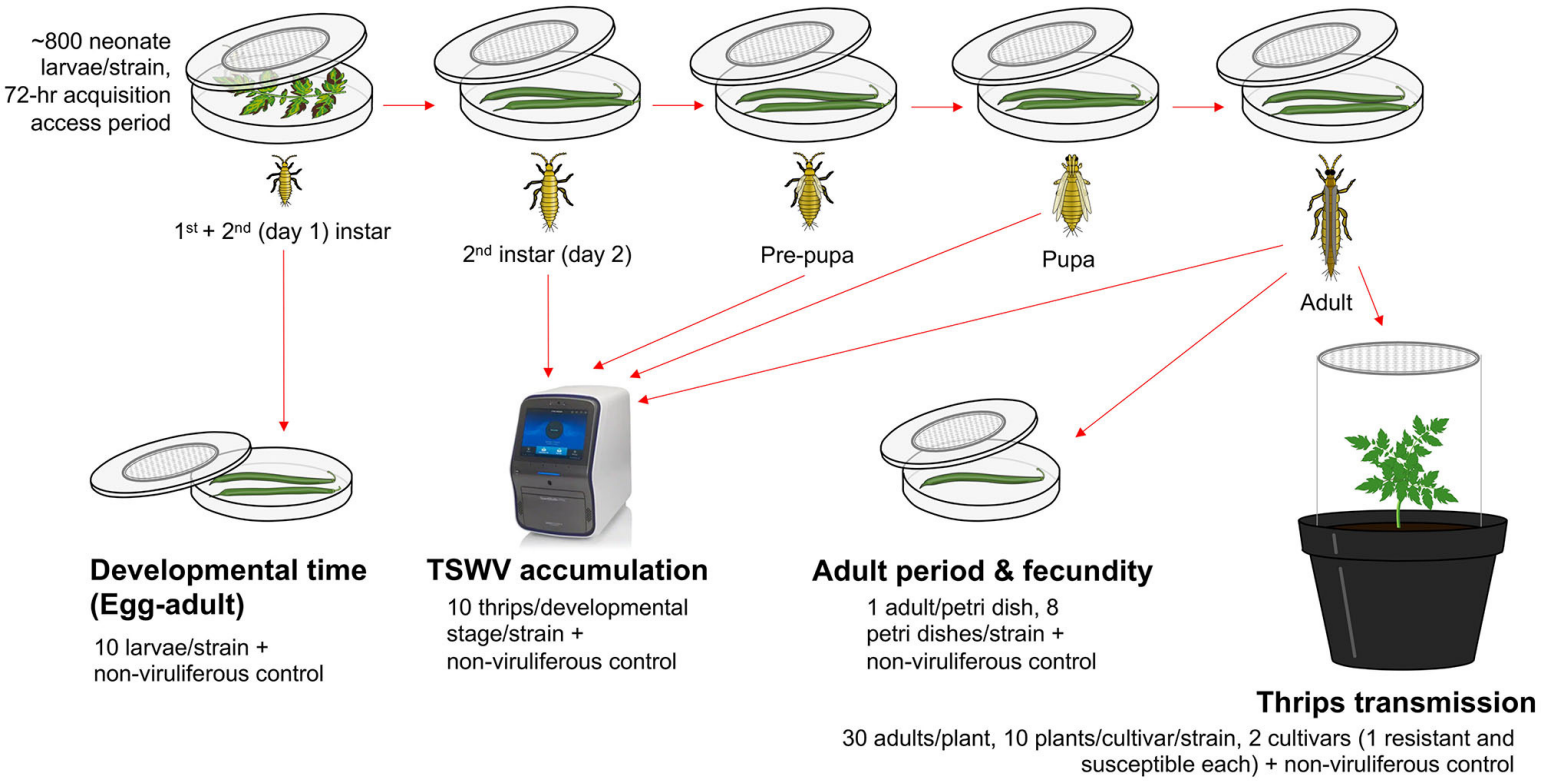
An overview of methods followed to study the transmission biology of resistance-breaking strains of TSWV. For each strain, ~800 neonate larvae were allowed a 72-h acquisition access period on non-infected tomato leaves or those infected with various TSWV strains to obtain non-viruliferous and viruliferous thrips, respectively. Different strains of TWSV used in the study were sympatric (from Bushland, TX): Tom-BL1, Tom-BL2; allopatric (from California) Tom-CA and (from Mexico) Tom-MX; and non-resistance breaking (non-RB). After virus acquisition, a cohort of 10 second instar thrips were transferred to a 9-cm sterile Petri dish (affixed with an insect mesh in the center) containing bean pods to record egg-to-adult developmental time every 12 h. Another large cohort of larvae was transferred to a different set of 14-cm Petri dishes through various developmental stages. At each stage, TSWV accumulation was quantified in 10 individual thrips per strain (and non-viruliferous control) using RT-qPCR. On the first day of adulthood, a subset of adult thrips was reared individually on 9-cm Petri dishes (one adult/Petri dish, eight Petri dishes/strain including control) to record the adult period and fecundity. To record fecundity, individual bean pods were replaced every 2 days with fresh ones. The collected old pods were then individually incubated in a new Petri dish for 3 days to record the number of emerging larvae. To study TSWV transmission, a large subset of larvae (30/plant) were allowed a 72-h inoculation access period on 3-week-old non-infect tomato plants from two different cultivars (one resistant and susceptible each, 10 plants/cultivar/strain) including non-viruliferous thrips as a control.

TSWV-infected tomato leaves expressing characteristic TSW symptoms (nearly equal symptom severity ratings of 4–5 on a scale of 5) from five strains with known TSWV titers (Tom-BL1 2 × 10^8^, Tom-BL2 1 × 10^8^, Tom-CA 6 × 10^6^, Tom-MX 5 × 10^5^, and non-RB 1 × 10^8^ copies/ng RNA) were placed in a separate 14-cm Petri dish (one each) with a lid containing insect-proof mesh at the center (Figure 1). Uninoculated healthy tomato leaves, with no TSWV infection, were used as a food source to obtain non-viruliferous thrips (negative control). For each strain, ~800 neonate first-instar larvae of WFT (3–4 h within hatching) were allowed to feed on these leaves with a 72-h acquisition access period (AAP). The larvae were then subsequently reared on green bean pods through adulthood. From the original 14-cm Petri dish, multiple cohorts of 10 thrips were randomly selected at each developmental stage to determine TSWV copy numbers using RT-qPCR. From the original 14-cm Petri dish, a subset of 10 larvae were moved to a 9-cm Petri dish (one per strain) immediately following the 72-h AAP along with non-viruliferous thrips from healthy control and allowed to feed on green bean pods to precisely record developmental time at each stage: egg through adult (till the first day of adulthood).

For each strain (and non-viruliferous control), eight female adult thrips developed from the original 14-cm Petri dish after rearing on green bean pods (on the first day of adulthood) were released in individual 9-cm plastic Petri dishes (one adult per Petri dish, eight Petri dishes per strain). The individual thrips were fed and allowed to oviposit on one green bean pod at a time. Old bean pods were replaced with fresh ones every 2 days. First, all subsequent old pods were individually incubated in a new Petri dish for 3 days at 25°C to count the number of emerging larvae as a measure of fecundity throughout the adult stage. For each adult, the number of days from the first day of adulthood through death was recorded as a measure of the adult period for each strain, including non-viruliferous control.

### 2.4. Thrips transmission

Transmission biology of WFT infected with different strains of TSWV was determined through TSWV incidence, symptom severity, and days till the onset of symptoms in two commercial tomato cultivars: one resistant and one susceptible. More specifically, 30 adults (a random mix of males and females) developed from the original 14-cm Petri dish after rearing on green bean pods (on the first day of adulthood) were released on five 3-week-old individually caged tomato plants from each cultivar (15 plants per strain) in insect-proof cages and allowed a 72-h inoculation access period (IAP) on those plants. Plants were then sprayed with a systemic insecticide to kill thrips and subsequently monitored for TSW symptom expression at 25°C during a 16-h photoperiod in a greenhouse. The severity of TSW symptoms was rated on a scale of 0–5, where 0 = no symptoms, 1 = mild mosaic on young leaves; 2 = mosaic and puckering of leaves; 3 = chlorotic spots and puckering of leaves; 4 = chlorotic and necrotic spots on leaves, bronzing, and stunting; and 5 = all symptoms in 4 plus partial wilting. Besides transmission parameters, TSWV titers in thrips-inoculated plants were measured 30 days post-inoculation (DPI).

### 2.5. RNA extraction and TSWV titers in individual thrips

To quantify the virus acquired by thrips from different TSWV strains, RNA was extracted from 10 individual thrips per strain at different developmental stages, including second instar, pre-pupae, pupae, and adult. Using a camel hairbrush, individual thrips were moved to 2-ml screw-cap tubes containing sterilized glass or metal beads. Tubes were vortexed in 20 μl of Quick Extract solution (Lucigen, Middleton, WI), followed by centrifugation at 12,000 rpm for 30 s. The tubes were then incubated at 65°C for 10 min followed by 98°C for 3 min. Immediately after heat shock, tubes were placed on ice for 5 min, briefly centrifuged, and the RNA suspended in the aqueous phase was transferred to a new tube and stored at −20°C for further use.

One-step RT-qPCR was used to detect and quantify TSWV. A volume of 20-μl reaction mixture contained 5 μl (4 × ) of TaqMan Fast Virus 1-Step Master Mix (Thermo Fisher Scientific, Waltham, MA, United States), 1 μl (10 pmol) of each nucleocapsid gene-specific forward (5′-AGAGCATAATGAAGGTTATTAAGCAAAGTGA-3′) and reverse primers (5′-GCCTGACCCTGATCAAGCTATC-3′), 1 μl (20 × ) of TaqMan probe ([6~FAM] CAGTGGCTCCAATCCT[BHQ1a~Q]), and 1 μl of RNA (50 ng). The mixture was analyzed in the Quant Studio 7 Pro system (Applied Biosystems, Waltham, MA, United States) with the following conditions: reverse transcription at 50°C for 10 min, holding at 94°C for 5 min, followed by 40 cycles of 94°C for 10 s and 60°C for 30 s. Absolute virus copy numbers were estimated using standards containing known copies of the qPCR-targeted TWSV coat protein gene. Standards were designed by Custom Applied Biosystems TaqMan Expression assays (Thermo Fisher Scientific, Waltham, MA, United States). Tenfold serial dilutions containing from 10^11^ to 10^1^ targeted gene copies were prepared using standards. RNA extracted from non-viruliferous thrips fed on non-infected tomato leaves was used as a negative control. A total of three technical replicates were analyzed in all RT-qPCR assays. All experiments were independently replicated three times.

### 2.6. Statistical analyses

Data from experimental repeats were pooled and analyzed in R version 3.6.0. Fecundity data were log-transformed to meet the assumptions of normality and homogeneity of variance before analysis using one-way ANOVA. Treatment means were compared with *post-hoc* Tukey's HSD tests using the “dplyr” package (Wickham et al., 2018). Adult period data and total developmental time in days (egg to adult) were analyzed using the Kruskal-Wallis test. The treatment medians were compared with a *post-hoc* Dunn's test. Stagewise developmental time (in days) and TSWV accumulation in thrips at different developmental stages were analyzed using a mixed-effect model in the “lme4” package, using strain and developmental stage as fixed effects and replications as a random effect (Bates et al., 2015). Similarly, virus accumulation in plants was analyzed using strains and the resistance status of the plant (susceptible or resistant) as fixed effects and replications as a random effect. For the mixed-effect model, treatment means were compared using the “glht” command in the “multcomp” package (Hothorn et al., 2008). To assess the functional relationship of TSWV copy numbers in thrips with infected tissues from which they acquired the virus and with thrips-inoculated plants used in transmission studies, regression analysis was performed. Statistical differences were considered significant at a *p*-value of < 0.05.

## 3. Results

### 3.1. Developmental time (egg to adult)

**Figure 2:**
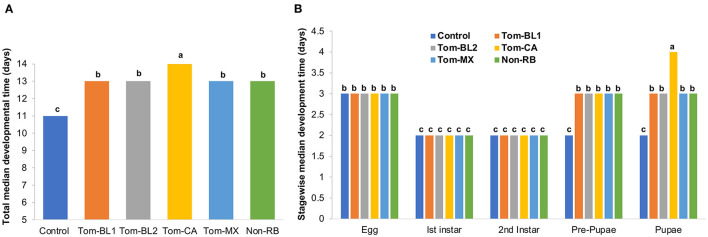
**(A)** Total and **(B)** stage-wise egg-to-adult developmental time of thrips individually infected with different strains of TWSV: Tom-BL1, Tom-BL2, Tom-CA, Tom-MX, and non-RB, with non-viruliferous control. Different letters indicate significant differences at *P* < 0.05.

TSWV infection in thrips did not affect the median developmental time of thrips immediately after virus acquisition, at the second instar stage, which lasted only 2 days. However, subsequent developmental stages, from pre-pupa through adulthood, were significantly affected. Total egg-to-adult (the first day of adulthood) developmental time differed significantly between viruliferous and non-viruliferous thrips [*F*_(5, 42)_ = 32.02; *P* < 0.01]. Overall, viruliferous thrips took a significantly longer time to develop from egg to adult compared to non-viruliferous thrips (Figure 2A). More specifically, the development of thrips infected with the Tom-CA strain was significantly slower compared to all other RB and non-RB strains and non-viruliferous control.

Stage-wise developmental time differed significantly across strains, especially in the late developmental stages [*F*_(29, 206)_ = 46.2; *P* < 0.01]. Across all strains and controls, the egg period was 3 days, whereas the first and second instar periods were 2 days each (Figure 2B). However, at the pre-pupal and pupal stages, all viruliferous thrips, regardless of the strain, showed significantly slower development compared to non-viruliferous controls. Across various strains, thrips infected with the Tom-CA strain took the longest time to develop from the pre-pupal stage to the pupal stage compared to thrips infected with other strains and controls.

### 3.2. Adult period and fecundity

**Figure 3:**
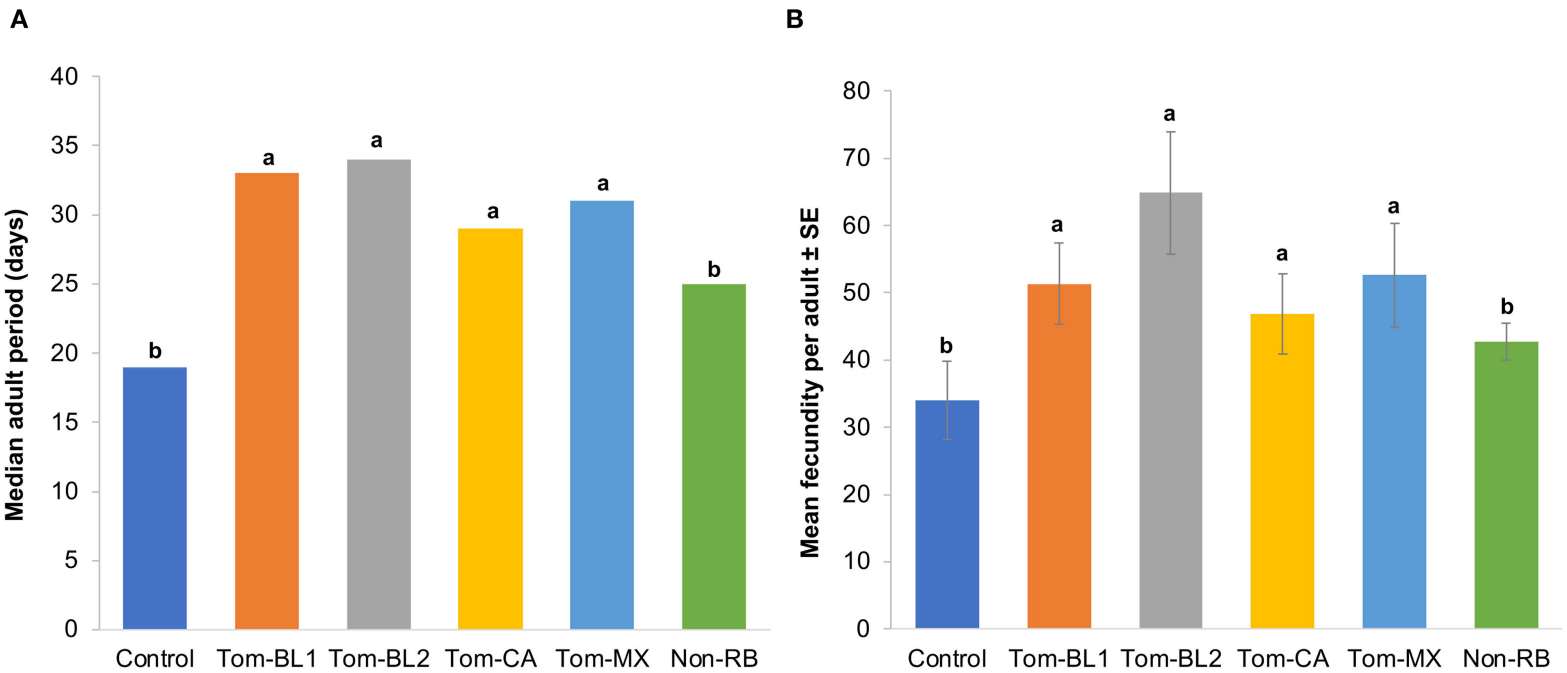
**(A)** Adult period and **(B)** fecundity of thrips individually infected with different strains of TWSV: Tom-BL1, Tom-BL2, Tom-CA, Tom-MX, and non-RB, with non-viruliferous control. Different letters indicate significant differences at *P* < 0.05.

Significant differences were observed in the adult period between treatments and the control [*F*_(5, 42)_ = 4.06; *P* = 0.004]. Interestingly, the adult period of both non-viruliferous thrips (18.5 days) and those infected with the non-RB strain (25 days) was significantly shorter compared to both sympatric (Tom-BL1 32.5; Tom-BL2 32.5 days) and allopatric (Tom-CA 28.5 days; Tom-MX 31 days) RB strains (Figure 3A). However, the adult period did not differ among various RB strains.

Consistent with an adult period, the number of offspring, a measure of fecundity produced by viruliferous and non-viruliferous thrips, was significantly different across different treatments [*F*_(5, 42)_ = 5.43; *P* < 0.006]. The fecundity of thrips infected with sympatric (Tom-BL1 51; Tom-BL2 65 offspring per adult) and allopatric (Tom-CA 47; Tom-MX 53 offspring per adult) RB strains was significantly higher compared to thrips infected with non-viruliferous (34 offspring/adult) and non-RB strains (43 offspring/adult) (Figure 3B). However, no differences in fecundity were observed within RB strains.

### 3.3. TSWV quantification in individual thrips at different developmental stages

**Figure 4:**
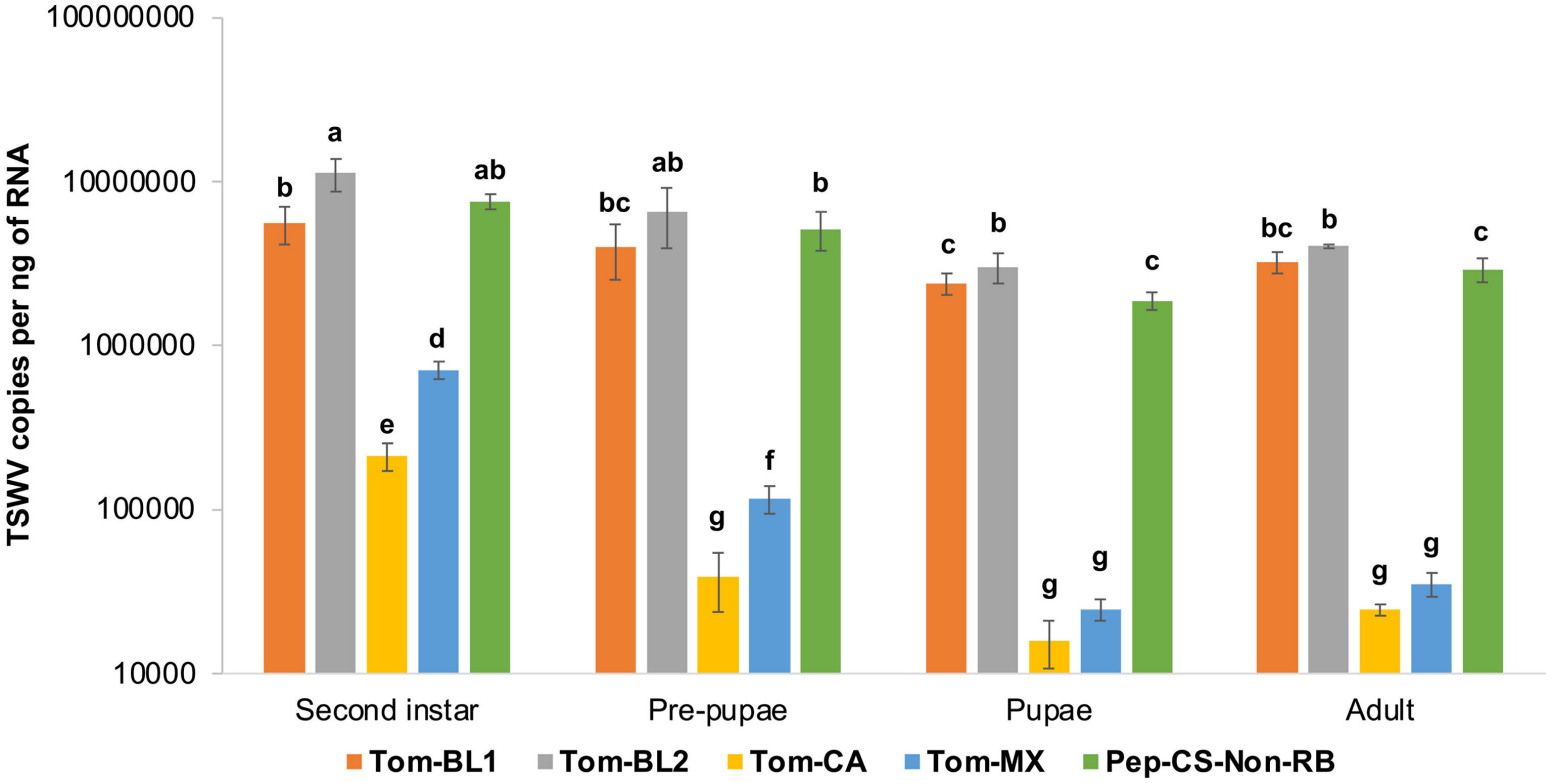
TWSV accumulation across different developmental stages of individual thrips infected with different strains of TWSV: Tom-BL1, Tom-BL2, Tom-CA, Tom-MX, and Non-RB, quantified using RT-qPCR. Different letters indicate significant differences at *P* < 0.05.

Neonate larvae from the first instar larval stage, which lasted for 2 days, followed by the first day of the second instar stage, were allowed to acquire TSWV for 72 h. Therefore, TSWV copy numbers in the first instar stage were not measured in the middle of virus acquisition. TSWV copies in thrips infected with Tom-BL1, Tom-BL2, and non-RB strains were significantly higher than those infected with Tom-MX and Tom-CA strains at all developmental stages [*F*_(19, 176)_ = 6.73; *P* < 0.001] (Figure 4). Of the strains, the accumulation of the RB strain Tom-CA was the lowest, especially at the first two time points, followed by the Tom-MX strain. Interestingly, Bushland RB strains, Tom-BL1 and Tom-BL2, accumulated at significantly different levels in thrips intermittently in the second instar and pupal stages when TSWV copies in the Tom-BL2 strain were significantly higher than those in the non-RB strain. At the second instar and pre-pupal stages, MX strain copies were higher than those of the CA strain but not in the subsequent developmental stages.

### 3.4. Thrips transmission

As expected, only RB-TSWV strains were able to infect *Sw-5b*-resistant tomato plants. However, TSW incidence, symptom severity, and days required for the first onset of symptoms were marginally different between strains (Table 1). Of the strains, TSW disease incidence in tomato plants was highest in both Bushland RB strains: 90% incidence in a resistant cultivar and 100% in a susceptible cultivar. For both strains, resistant plants showed TSWV symptoms at 34–35 DPI, whereas susceptible plants expressed symptoms quickly at 27 DPI. However, TSWV symptom severity was similar for both strains (3–4) in both resistant and susceptible cultivars. TSWV incidence in Mexican and California RB strains was 80% in a resistant cultivar, whereas it was 100% in a susceptible cultivar. However, the times required for symptom expression in resistant and susceptible cultivars were shorter in the Mexican strain (31 and 27 DPI) compared to the CA strain (37 and 30 DPI), respectively. While the degree of severity of symptoms did not vary between resistant and susceptible cultivars for the Mexican strain, it was marginally less severe in resistant plants (3) compared to susceptible cultivars (5) for the California strain. Finally, TSW incidence in a non-RB-infected susceptible tomato cultivar was 80%, with a high degree of symptom severity (5).

**Table 1:** Percentage of TSW incidence, symptom severity, and the onset of symptom expression in thrips-transmitted resistant and susceptible plants infected with five TSWV strains.

**TSWV isolates**	**Cultivars**	**% TSW incidence**	**Median TSW severity**	**Days till symptom expression**
Tom- BL1	Resistant	90	4	35
	Susceptible	100	4	27
Tom- BL2	Resistant	90	3	34–35
	Susceptible	100	4	27
Tom-CA	Resistant	80	3	37
	Susceptible	100	5	30
Tom- MX	Resistant	80	4	31
	Susceptible	100	4	27
Non-RB	Resistant	0	–	–
	Susceptible	80	5	25

**Figure 5:**
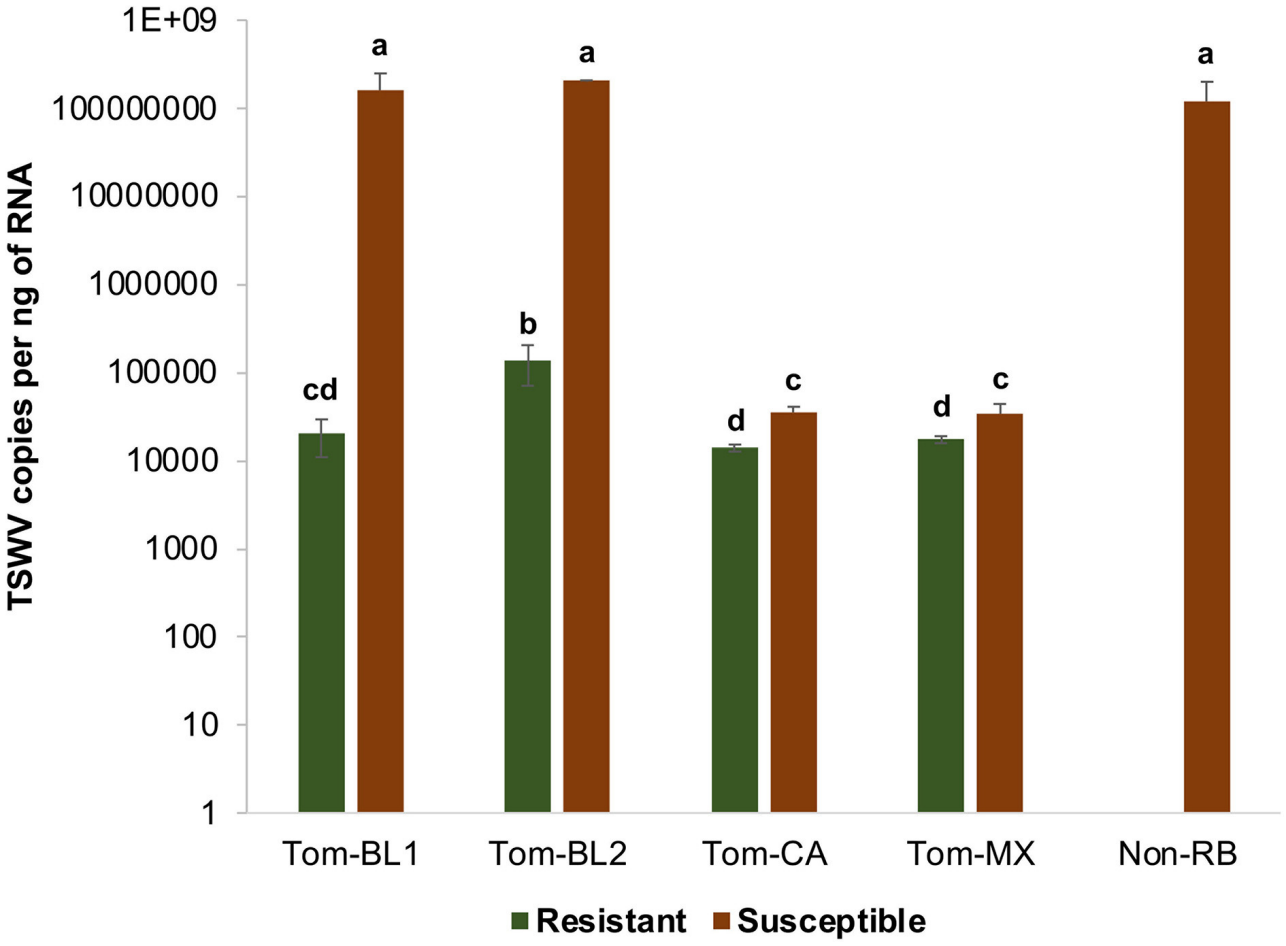
TSWV accumulation in thrips-inoculated tomato plants infected with different strains of TWSV: Tom-BL1, Tom-BL2, Tom-CA, Tom-MX, and Non-RB. TSWV was quantified 4 weeks post-inoculation from the topmost leaf using RT-qPCR. Different letters indicate significant differences at *P* < 0.05.

**Figure 6:**
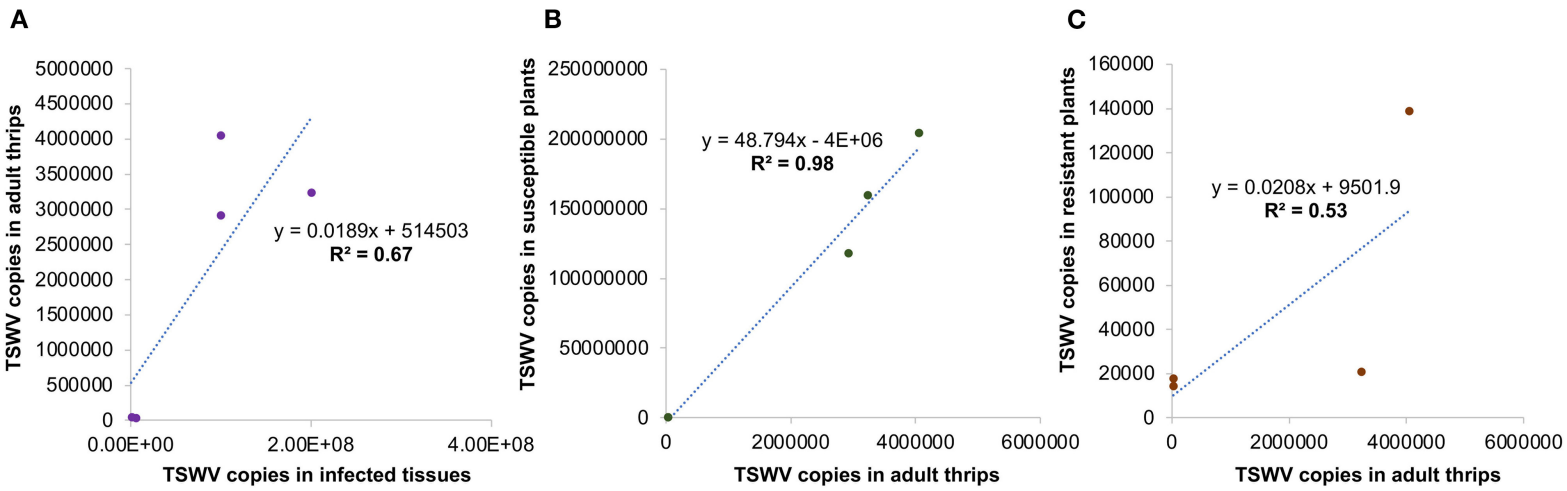
Regression analysis to assess functional relationships between TSWV copy numbers in thrips with those in **(A)** infected tissues from which they acquired the virus and thrips-inoculated, **(B)** susceptible, and **(C)** resistant plants used in transmission studies.

Significant differences in TSWV copies were observed in plants infected with different TSWV strains [*F*_(8, 81)_ =38.4; *P* < 0.001]. All strains accumulated in significantly higher amounts in susceptible plants than in resistant plants (Figure 5). Susceptible cultivars infected with Bushland RB and non-RB strains had significantly higher TSWV copies than the susceptible plants infected with Tom-CA and Tom-MX strains. Bushland strain Tom-BL2 accumulated in significantly higher numbers in resistant plants than all other RB strains. Regression analysis showed that TSWV copy numbers in adult thrips were not significantly related to those in TSWV-infected plant tissues from which thrips acquired the virus (*F* = 6.12; *P* = 0.089; *r*^2^ = 0.67) (Figure 6A). However, a strong positive correlation was observed between TSWV copy numbers in adults with those in thrips-transmitted susceptible plants (*F* = 178.2; *P* < 0.001; *r*^2^ = 0.98) (Figure 6B), but not the resistant ones (*F* = 2.21; *P* = 0.27; *r*^2^ = 0.52) (Figure 6C).

## 4. Discussion

Critical steps in broadening our current understanding of insect vector-plant pathogen interactions involve investigating how new strains of generalist viruses adapt to hosts, insect vectors, and the environment. We found that both sympatric (Tom-BL1 and Tom-BL2) and allopatric (Tom-CA and Tom-MX) novel resistance-breaking strains of TSWV benefit thrips by prolonging the adult period—arguably the most consequential developmental stage of thrips and by increasing fecundity—a major determinant of the reproductive success of insects in general. WFT-transmitted resistant and susceptible plants exhibit a higher incidence of TSW in RB strains compared to non-RB strains. This is the first extensive study on the transmission biology of TSWV RB strains and presents novel RB strains-mediated increased fitness of thrips and incidence of TSW in thrips-transmitted plants as one of the plausible explanations for the increased prevalence of RB strains worldwide, besides tremendous selection pressure on TSWV in the form of *Sw-5b*-resistant cultivars.

Interestingly, in non-viruliferous thrips and those infected with non-RB strains, the adult period was significantly shorter, and fecundity was significantly higher than in thrips infected with RB strains, regardless of their origin. Our findings partly align with prior studies, in that TSWV infection was reported to prolong developmental time (Ogada et al., 2013; Wan et al., 2020) and increase fecundity (Nachappa et al., 2020). However, because the non-RB strain in the present study was unable to affect either of the life history traits, there is a partial contrast to these prior studies, which appear to have used local or wild-type TSWV strains. The observed differences could be attributed to differences in experimental design, hosts, or strains.

Overall, across all strains, TSWV copy numbers were high in the second instar but declined significantly at the pupal stage, followed by a slight, non-significant increase at the adult stage. This is consistent with thrips developmental physiology, in that TSWV replication in thrips peaked during active feeding and metabolic stages, second instar larva and adult, as opposed to non-feeding and metabolically less active stages, pre-pupa and pupa. TSWV copies in individual thrips varied significantly across strains and developmental stages. For the most part, this study shows harmony with previous findings by Linak et al. (2020), in which TSWV titers in the vector *Thrips tabaci* were found to be unrelated to virus titers in the leaf tissue from which they acquired the virus. Despite drastic variations in copy numbers in thrips adults (lowest copies in CA and MX strains), TSW incidence was higher (100%) in RB-infected susceptible plants compared to the non-RB-infected ones (80%). However, in a resistant cultivar, the TSW incidence of sympatric strains was marginally higher (90%) than the allopatric ones (80%).

A significant and consistent positive association between the frequency of transmission and virus titer was reported in a prior study by Rotenberg et al. (2009). Similarly, our study showed that the virus titer in adult thrips was strongly correlated to the virus titer in thrips-inoculated susceptible plants. For instance, high virus titers in plants infected with Bushland and non-RB strains were significantly positively correlated to those in adult thrips. However, this correlation was significant only in susceptible plants but not in the resistant ones, possibly due to comparatively lower TSWV copy numbers in resistant plants. Regardless of the virus copy numbers, TSW symptoms severity was nearly similar in all RB-infected resistant and susceptible plants, except for the California strain, in which symptoms appeared to be marginally more severe in susceptible as opposed to plants. Furthermore, in all RB strains, TSW symptoms appeared in susceptible plants a week faster than in resistant plants, except for the Mexican strain, where the difference was only 4 days. The fastest symptom development was observed in susceptible plants infected with a non-RB strain (25 days), possibly due to the high virus titer in these plants. However, no robust association of symptom severity with virus titer in infected plants was observed in other strains.

Several factors have been reported to determine the successful transmission of TSWV by thrips. At the molecular level, specific interactions of TSWV proteins such as silencing suppressor (NSs), nucleocapsid (N), and glycoproteins (G_N_/G_C_) with TSWV-interacting proteins (TIPs) of thrips and host resistance genes of plants have been documented to facilitate TSWV infection and movement in plant and insect hosts (Ullman et al., 1993; Hallwass et al., 2014; Montero-Astúa et al., 2014; Badillo-Vargas et al., 2019). Furthermore, thrips' mode of reproduction and sex has been documented to be one of the major determinants of TSWV transmission. For instance, arrhenotokous (sexually reproducing) populations of thrips and males have been reported to be more efficient at transmitting TSWV than thelytokous (parthenogenetic) populations and females (Chatzivassiliou et al., 2002; Rotenberg et al., 2009). Since all thrips used in our study were randomly selected from a large single-synchronous population, it is unlikely that either of these parameters would have affected our findings. Indeed, it would be interesting to understand whether and how these factors determine the transmission efficiency of RB strains.

The selection pressure imposed by the worldwide deployment of *Sw-5b*-resistant tomato and *Tsw*-resistant pepper cultivars on TSWV is the single most important factor in the recent global emergence of resistance-breaking strains worldwide. Our findings provide novel insights into how the mutually beneficial intimate association between TSWV and thrips may offer epidemiological advantage to RB strains through increased vector fitness and further contribute to a re-emerging pandemic of TSWV. An integrative analysis of novel RB strains is needed to systematically investigate molecular mechanisms underpinning TSWV infection, transmission, and their interactions with cross-kingdom hosts and to devise sustainable pest management strategies.

## Data availability statement

The datasets presented in this study can be found in online repositories. The names of the repository/repositories and accession number(s) can be found in the article/supplementary material.

## Author contributions

SC: Data curation, Investigation, Methodology, Visualization, Writing—original draft, Writing—review and editing. SG: Data curation, Methodology, Visualization, Writing—review and editing, Conceptualization, Formal analysis, Software, Validation. BH: Data curation, Methodology, Visualization, Writing—review and editing, Investigation. FW: Writing—review and editing, Funding acquisition, Resources, Supervision, Validation. CR: Funding acquisition, Resources, Writing—review and editing, Conceptualization. KG: Conceptualization, Funding acquisition, Resources, Writing—review and editing, Data curation, Investigation, Methodology, Project administration, Supervision, Validation, Visualization, Writing—original draft.

## References

[B1] Adkins S. Quadt R. Choi T.-J. Ahlquist P. German T. (1995). An RNA-dependent RNA polymerase activity associated with virions of tomato spotted wilt virus, a plant- and insect-infecting bunyavirus. Virology 207, 308–311. 10.1006/viro.1995.1083 7871744

[B2] Almási A. Pinczés D. Tímár Z. Sáray R. Palotás G. Salánki K. (2023). Identification of a new type of resistance breaking strain of tomato spotted wilt virus on tomato bearing the Sw-5b resistance gene. Eur. J. Plant Pathol. 166, 219–225. 10.1007/s10658-023-02656-5

[B3] Aramburu J. Marti M. (2003). The occurrence in north-east Spain of a variant of Tomato spotted wilt virus (TSWV) that breaks resistance in tomato (*Lycopersicon esculentum*) containing the Sw-5 gene. Plant Pathol. 52, 407–407. 10.1046/j.1365-3059.2003.00829.x

[B4] Badillo-Vargas I. E. Chen Y. Martin K. M. Rotenberg D. Whitfield A. E. (2019). Discovery of novel thrips vector proteins that bind to the viral attachment protein of the plant bunyavirus tomato spotted wilt virus. J. Virol. 93, 10–1128. 10.1128/JVI.00699-19 31413126 PMC6803271

[B5] Bates D. Mächler M. Bolker B. Walker S. (2015). Fitting linear mixed-effects models using lme4. J. Stat. Softw. 67, 1–48. 10.18637/jss.v067.i01

[B6] Batuman O. Turini T. A. Oliveira P. V. Rojas M. R. Macedo M. Mellinger H. C.. (2017). First report of a resistance-breaking strain of Tomato spotted wilt virus infecting tomatoes with the *Sw-*5 tospovirus-resistance gene in California. Plant Dis. 101, 637–637. 10.1094/PDIS-09-16-1371-PDN

[B7] Belliure B. Janssen A. Maris P. C. Peters D. Sabelis M. W. (2004). Herbivore arthropods benefit from vectoring plant viruses. Ecol. Lett. 8, 70–79. 10.1111/j.1461-0248.2004.00699.x

[B8] Boiteux L. S. (1995). Allelic relationships between genes for resistance to tomato spotted wilt tospovirus in *Capsicum chinense*. Theoret. Appl. Genet. 90, 146–149. 10.1007/BF00221009 24173797

[B9] Boiteux L. S. De Avila A. C. (1994). Inheritance of a resistance specific to tomato spotted wilt tospovirus in *Capsicum chinensè* PI 159236'. Euphytica 75, 139–142. 10.1007/BF00024541

[B10] Chatzivassiliou E. K. Nagata T. Katis N. I. Peters D. (1999). Transmission of tomato spotted wilt tospovirus by *Thrips tabaci* populations originating from leek. Plant Pathol. 48, 700–706. 10.1046/j.1365-3059.1999.00414.x

[B11] Chatzivassiliou E. K. Peters D. Katis N. I. (2002). The efficiency by which *Thrips tabaci* populations transmit Tomato spotted wilt virus depends on their host preference and reproductive strategy. Phytopathology 92, 603–609. 10.1094/PHYTO.2002.92.6.603 18944256

[B12] Chinnaiah S. Gautam S. Workneh F. Crosby K. Rush C. Gadhave K. R. (2023). First report of Sw-5 resistance-breaking strain of tomato spotted wilt orthotospovirus infecting tomato in Texas. Plant Dis. 10.1094/PDIS-11-22-2699-PDN36916839

[B13] Ciuffo M. Finetti-Sialer M. M. Gallitelli D. Turina M. (2005). First report in Italy of a resistance-breaking strain of Tomato spotted wilt virus infecting tomato cultivars carrying the Sw5 resistance gene. Plant Pathol. 54, 564–564. 10.1111/j.1365-3059.2005.01203.x

[B14] De Angelis J. D. Sether D. M. Rossignol P. A. (1993). Survival, development, and reproduction in western flower thrips (Thysanoptera: Thripidae) exposed to impatiens necrotic spot virus. Environ. Entomol. 22, 1308–1312. 10.1093/ee/22.6.1308

[B15] De Oliveira A. S. Boiteux L. S. Kormelink R. Resende R. O. (2018). The Sw-5 gene cluster: Tomato breeding and research toward orthotospovirus disease control. Front Plant Sci. 9, 1055. 10.3389/fpls.2018.01055 30073012 PMC6060272

[B16] Dianese E. C. Fonseca M. E. N. Inoue-Nagata A. K. Resende R. O. Boiteux L. S. (2011). Search in *Solanum* (section *Lycopersicon*) germplasm for sources of broad-spectrum resistance to four *Tospovirus species*. Euphytica 180, 307–319. 10.1007/s10681-011-0355-8

[B17] Fidan H. Sari N. (2019). Molecular characterization of resistance-breaking Tomato spotted wilt virus (TSWV) isolate medium segment in tomato. Appl. Ecol. Environ. Res. 17, 5321–5339. 10.15666/aeer/1702_53215339

[B18] Gautam S. Chinnaiah S. Workneh F. Crosby K. Rush C. Gadhave K. R. (2023). First report of a resistance-breaking strain of tomato spotted wilt orthotospovirus infecting *Capsicum annuum* with the *Tsw* resistance gene in Texas. *Plant Dis*. 10.1094/PDIS-09-22-2274-PDN36383996

[B19] Hallwass M. De Oliveira A. S. de Campos Dianese E. Lohuis D. Boiteux L. S. Inoue-Nagata A. K.. (2014). The Tomato spotted wilt virus cell-to-cell movement protein (NSM) triggers a hypersensitive response in Sw-5-containing resistant tomato lines and in *Nicotiana benthamiana* transformed with the functional Sw-5b resistance gene copy. Mol. Plant Pathol. 15, 871–880. 10.1111/mpp.12144 24720811 PMC6638845

[B20] Hothorn T. Bretz F. Westfall P. (2008). Simultaneous inference in general parametric models. Biom. J. 50, 346–363. 10.1002/bimj.200810425 18481363

[B21] Lahre K. A. Shekasteband R. Meadows I. Whitfield A. E. Rotenberg D. (2023). First report of resistance-breaking variants of tomato spotted wilt virus (TSWV) infecting tomatoes with the Sw-5 tospovirus-resistance gene in North Carolina. Plant Dis. 10.1094/PDIS-11-22-2637-PDN36627809

[B22] Linak J. A. Jacobson A. L. Sit T. L. Kennedy G. G. (2020). Relationships of virus titers and transmission rates among sympatric and allopatric virus isolates and thrips vectors support local adaptation. Sci. Rep. 10, 7649. 10.1038/s41598-020-64507-1 32376869 PMC7203134

[B23] Macedo M. A. Rojas M. R. Gilbertson R. L. (2019). First report of a resistance-breaking strain of tomato spotted wilt orthotospovirus infecting sweet pepper with the *Tsw* resistance gene in California, U.S.A. Plant Dis. 103, 1048. 10.1094/PDIS-07-18-1239-PDN

[B24] Margaria P. Ciuffo M. Turina M. (2004). Resistance breaking strain of Tomato spotted wilt virus (Tospovirus; Bunyaviridae) on resistant pepper cultivars in Almeria, Spain. Plant Pathol. 53, 795–795. 10.1111/j.1365-3059.2004.01082.x

[B25] Margaria P. Ciuffo M. Turina M. (2007). Evidence that the nonstructural protein of Tomato spotted wilt virus is the avirulence determinant in the interaction with resistant pepper carrying the TSW gene. Mol. Plant Microbe Interact. 20, 547–558 10.1094/MPMI-20-5-0547 17506332

[B26] Maris P. C. Joosten N. N. Goldbach R. W. Peters D. (2004). *Tomato spotted wilt virus* infection improves host suitability for its vector *Frankliniella occidentalis*. Phytopathology 94, 706–711. 10.1094/PHYTO.2004.94.7.706 18943902

[B27] Montero-Astúa M. Rotenberg D. Leach-Kieffaber A. Schneweis B. A. Park S. Park J. K.. (2014). Disruption of vector transmission by a plant-expressed viral glycoprotein. Mol. Plant Microbe Interact. 27, 296–304. 10.1094/MPMI-09-13-0287-FI 24405031

[B28] Nachappa P. Challacombe J. Margolies D. C. Nechols J. R. Whitfield A. E. Rotenberg D. (2020). Tomato spotted wilt virus benefits its thrips vector by modulating metabolic and plant defense pathways in tomato. Front. Plant Sci. 11, 575564. 10.3389/fpls.2020.575564 33424878 PMC7793759

[B29] Ogada P. A. Maiss E. Poehling H.-M. (2013). Influence of *tomato spotted wilt virus* on performance and behaviour of western flower thrips (*Frankliniella occidentalis*). J. Appl. Entomol. 137, 488–498. 10.1111/jen.12023

[B30] Pappu H. R. Jones R. A. C. Jain R. K. (2009). Global status of tospovirus epidemics in diverse cropping systems: successes achieved and challenges ahead. Virus Res. 141, 219–236. 10.1016/j.virusres.2009.01.009 19189852

[B31] Peiró A. Cañizares M. C. Rubio L. López C. Moriones E. Aramburu J.. (2014). The movement protein (NSm) of Tomato spotted wilt virus is the avirulence determinant in the tomato Sw-5 gene-based resistance. Mol. Plant Pathol. 15, 802–813. 10.1111/mpp.12142 24690181 PMC6638753

[B32] Rotenberg D. Krishna Kumar N. K. Ullman D. E. Montero-Astúa M. Willis D. K. German T. L.. (2009). Variation in *Tomato spotted wilt virus* titer in *Frankliniella occidentalis* and its association with frequency of transmission. Phytopathology 99, 404–410. 10.1094/PHYTO-99-4-0404 19271982

[B33] Scholthof K.-B. G. Adkins S. Czosnek H. Palukaitis P. Jacquot E. Hohn T.. (2011). Top 10 plant viruses in molecular plant pathology. Mol. Plant Pathol. 12, 938–954. 10.1111/j.1364-3703.2011.00752.x 22017770 PMC6640423

[B34] Shalileh S. Ogada P. A. Moualeu D. P. Poehling H.-M. (2016). Manipulation of *Frankliniella occidentalis* (Thysanoptera: Thripidae) by *Tomato Spotted Wilt Virus* (Tospovirus) via the host plant nutrients to enhance its transmission and spread. Environ. Entomol. 45, 1235–1242. 10.1093/ee/nvw102 27566527 PMC5037971

[B35] Sharman M. Persley D. M. (2006). Field isolates of Tomato spotted wilt virus overcoming resistance in capsicum in Australia. Aust. Plant Pathol. 35, 123–128. 10.1071/AP06014

[B36] Shrestha A. Srinivasan R. Riley D. G. Culbreath A. K. (2012). Direct and indirect effects of a thrips-transmitted *Tospovirus* on the preference and fitness of its vector, *Frankliniella fusca*. Entomol. Exp. Appl. 145, 260–271. 10.1111/eea.12011

[B37] Stumpf C. F. Kennedy G. G. (2005). Effects of tomato spotted wilt virus (TSWV) isolates, host plants, and temperature on survival, size, and development time of *Frankliniella fusca*. Entomol. Exp. Appl. 114, 215–225. 10.1111/j.1570-7458.2005.00251.x

[B38] Ullman D. E. German T. L. Sherwood J. L. Westcot D. M. Cantone F. A. (1993). Tospovirus replication in insect vector cells: immunocytochemical evidence that the nonstructural protein encoded by the S RNA of tomato spotted wilt tospovirus is present in thrips vector cells. Phytopathology 83, 456–463. 10.1094/Phyto-83-456

[B39] Wan Y. Hussain S. Merchant A. Xu B. Xie W. Wang S.. (2020). Tomato spotted wilt orthotospovirus influences the reproduction of its insect vector, western flower thrips, *Frankliniella occidentalis*, to facilitate transmission. Pest Manag. Sci. 76, 2406–2414. 10.1002/ps.5779 32030849

[B40] Whitfield A. E. Ullman D. E. German T. L. (2005). Tospovirus-thrips interactions. Annu. Rev. Phytopathol. 43, 459–489. 10.1146/annurev.phyto.43.040204.140017 16078892

[B41] Wickham H. François R. Henry L. Müller K. (2018). dplyr: A Grammar of Data Manipulation. R Package Version 0.7. 6. Computer software. Available online at: https://CRAN.R-project.org/package=dplyr (accessed July 12, 2023).

[B42] Yoon J. Y. Her N. H. Cho I. S. Chung B. N. Choi S. K. (2021). First report of a resistance-breaking strain of Tomato spotted wilt orthotospovirus infecting *Capsicum annuum* carrying the Tsw resistance gene in South Korea. Plant Dis. 105, 2259. 10.1094/PDIS-09-20-1952-PDN33591834

[B43] Zaccardelli M. Perrone D. Del Galdo A. Campanile F. Parrella G. Giordano I. (2008). Tomato genotypes resistant to tomato spotted wilt virus evaluated in open field crops in Southern Italy. Acta Hortic. 789, 147–150. 10.17660/ActaHortic.2008.789.20

